# Hemodynamic and behavioral changes in older adults during cognitively demanding dual tasks

**DOI:** 10.1002/brb3.2021

**Published:** 2021-01-08

**Authors:** Talia Salzman, Diana Tobón Vallejo, Nadia Polskaia, Lucas Michaud, Gabrielle St‐Amant, Yves Lajoie, Sarah Fraser

**Affiliations:** ^1^ Interdisciplinary School of Health Sciences University of Ottawa Ottawa ON Canada; ^2^ Faculty of Engineering Universidad de Medellín Medellín Colombia; ^3^ School of Human Kinetics University of Ottawa Ottawa ON Canada

**Keywords:** Aging, Cognitive science, dual‐task walking, executive demands, fNIRS, Gait, Near Infra‐red spectroscopy, Task Performance and Analysis

## Abstract

**Introduction:**

Executive functions play a fundamental role in walking by integrating information from cognitive‐motor pathways. Subtle changes in brain and behavior may help identify older adults who are more susceptible to executive function deficits with advancing age due to prefrontal cortex deterioration. This study aims to examine how older adults mitigate executive demands while walking during cognitively demanding tasks.

**Methods:**

Twenty healthy older adults (*M = *71.8 years, *SD* = 6.4) performed simple reaction time (SRT), go/no‐go (GNG), n‐back (NBK), and double number sequence (DNS) cognitive tasks of increasing difficulty while walking (i.e., dual task). Functional near infra‐red spectroscopy (fNIRS) was used to measure the hemodynamic response (i.e., oxy‐ [HbO2] and deoxyhemoglobin [HbR]) changes in the prefrontal cortex (PFC) during dual and single tasks (i.e., walking alone). In addition, performance was measured using gait speed (m/s), response time (s), and accuracy (% correct).

**Results:**

Using repeated measures ANOVAs, neural findings demonstrated a main effect of task such that ∆HbO2 (*p* = .047) and ∆HbR (*p* = .040) decreased between single and dual tasks. An interaction between task and cognitive difficulty (*p* = .014) revealed that gait speed decreased in the DNS between single and dual tasks. A main effect of task in response time indicated that the SRT response time was faster than all other difficulty levels (*p* < .001). Accuracy performance declined between single and dual tasks (*p* = .028) and across difficulty levels (*p* < .001) but was not significantly different between the NBK and DNS.

**Conclusion:**

Findings suggest that a healthy older adult sample might mitigate executive demands using an automatic locomotor control strategy such that shifting conscious attention away from walking during the dual tasks resulted in decreased ∆HbO2 and ∆HbR. However, decreased prefrontal activation was inefficient at maintaining response time and accuracy performance and may be differently affected by increasing cognitive demands.

## Graphical abstract

Older adults may experience executive function deficits due to prefrontal cortex (PFC) deterioration. Findings revealed decreased PFC activation between single and dual tasks and worse cognitive performance (i.e., slower response times and lower accuracy scores) with increasing cognitive task demands. However, gait speed was maintained until the most demanding cognitive task after which it declined between single and dual tasks.

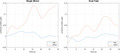

## 1 INTRODUCTION

Declines in cognition are more common as people age and have been supported by studies examining changes in brain activation between older and younger adults (Fraser et al., 2016; Grady, 2000; Holtzer et al., 2011). Neuroimaging findings suggest that compensatory neural mechanisms exist to counteract decline and to allow for the maintenance of cognition over time (Cabeza et al., 2018; Reuter‐Lorenz & Park, 2014). One example is the revised Scaffolding Theory of Aging and Cognition (STAC‐r) which outlines compensatory scaffolding as an adaptive measure for older adults to generate and recruit additional neural resources to replace those that have deteriorated over time (Reuter‐Lorenz & Park, 2014). A variety of cognitive tasks have been used to examine this phenomenon including working memory and serial subtraction tasks (Pelicioni et al., 2019). Working memory studies have revealed that older adults exhibit increased, and bilateral brain activation compared to younger adults (Fraser et al., 2016; Vermeij et al., 2012). However, when manipulating the difficulty of a working memory (Fraser et al., 2016) or serial subtraction (Mirelman et al., 2017) task, greater increases were observed with increasing cognitive task difficulty. In contrast, when a cognitive task insufficiently challenged older adults, brain activation changes between age groups were insignificant (Marusic et al., 2019). Therefore, STAC‐r may account for greater brain activation changes in older versus younger adults when behavioral measures are similar between both groups. More evidence is needed to determine which types of cognitive tasks may differentially affect or challenge older adults and result in changes in brain activity with or without corresponding changes in performance.

Behavioral measures of performance such as gait speed have also been used to evaluate cognition (Al‐Yahya et al., 2011). Early research has demonstrated that some older adults are unable to walk and talk at the same time and those that stopped walking to talk were more prone to falling (Lundin‐Olsson et al., 1997). While walking alone did not lead to any gait changes, slowing down or stopping may be an involuntary strategy exhibited by older adults to prioritize gait and ensure safe ambulation (Holtzer et al., 2016; Shumway‐Cook et al., 1997). Alternatively, higher functioning and cognitively healthy older adults may resemble younger adults in that they exhibit an automatic locomotor control strategy to manage walking and talking simultaneously (i.e., dual‐tasking) (Bernstein, 1967). Automatic control is efficient in that steady state walking can be achieved under minimal conscious attention thereby freeing up executive resources for a secondary task (Clark, 2015; Poldrack, 2005). However, studies have demonstrated that greater task difficulty may lead to a loss of automaticity and greater reliance on the prefrontal cortex (PFC) due to the attentional demands associated with maintaining gait performance (Clark, 2015; Holtzer et al., 2015). This is known as the executive control of walking, which operates under a limited processing capacity, but may be recruited when dual tasks require greater executive resources (Beurskens & Bock, 2012; Yogev et al., 2008).

The PFC is responsible for mediating complex cognitive processes, namely, planning, attention, and coordination, which are involved in everyday tasks such as walking or dual‐tasking (Cabeza et al., 2018). In fact, the dual‐task paradigm measures changes in executive functioning by comparing brain activation and performance between single and dual tasks (Pashler, 1994). Reviews in the literature demonstrate inconsistent findings as to whether prefrontal activation and behavior should increase, decrease, or stay the same between single and dual tasks (Kahya et al., 2019; Pelicioni et al., 2019). This may be due to diverse cognitive tasks such as verbal fluency (Hawkins et al., 2018; Holtzer et al., 2015; Verghese et al., 2017) and counting backwards (Al‐Yahya et al., 2011; Mirelman et al., 2017) which differentially engage executive functions and the PFC. Therefore, it may important to account for differences in cognitive task difficulty between studies (Patel et al., 2014). One approach to mitigate this concern is a study design that targets the examination of executive functioning across multiple task difficulties. This may also allow for the identification of easier cognitive tasks that are not sensitive enough or do not challenge older adults sufficiently to detect changes in single versus dual tasks. More specifically, this may reveal whether executive control is only evoked under greater cognitive demands and whether STAC‐r compensatory mechanisms are efficient enough to preserve performance.

In order to simultaneously examine the neural and behavioral mechanisms underlying executive functioning, functional near infra‐red spectroscopy (fNIRS) can be used to monitor cerebral oxygenation (∆HbO2) and deoxygenation (∆HbR) changes in the PFC. FNIRS is advantageous over other functional neuroimaging techniques most notably for its noninvasive and portable nature that does not limit an individual's mobility (Pinti et al., 2018). In its application to walking, it tolerates motion artifacts better than other techniques and can be used on people of all ages with no adverse health consequences (Pinti et al., 2018). FNIRS exploits the transient nature of biological tissue to near‐infrared light as well as the distinct absorption spectra of oxygenated (HbO2) and deoxygenated (HbR) hemoglobin in the near‐infrared region (Quaresima & Ferrari, 2019). In theory, the PFC requires an influx of HbO2 and efflux of HbR as cognitive demands increase. Therefore, during dual tasks, the increased cerebral blood flow and metabolic demand of oxygen can be coupled in a process known as neurovascular coupling (Quaresima & Ferrari, 2019). This process can then serve as a neurophysiological marker for fNIRS to detect changes in cerebral oxygenation during dual‐task walking studies (Al‐Yahya et al., 2016; Sorond et al., 2011).

Furthermore, various behavioral measures can be used to quantify the shift from performance maintenance to decline. First, gait speed is a commonly used measure to assess locomotor control (Hausdorff et al., 2008; Smith et al., 2016; Yogev et al., 2008). Studies have demonstrated a strong relationship between poor executive functioning and slower gait speed especially during dual tasks involving a challenging locomotor component (Hawkins et al., 2018; Maidan et al., 2016; Mirelman et al., 2017). This is in line with the executive processing of gait which is recruited when tasks are unlearned or too challenging to be automatically processed (Clark, 2015). Other behavioral measures such as response time and accuracy have been reported in the literature but with greater variability across different task types and difficulty levels. For example, slower response times were observed between single and dual tasks during a cognitive‐auditory task, but there were no differences in accuracy (Rosso et al., 2017). In contrast, studies examining neural inhibition and working memory have demonstrated that cognitive performance declines in older adults in dual compared to single tasks (Fraser et al., 2016; Hsieh et al., 2016). This may be due to the complex processing steps involved in discerning between relevant and irrelevant stimuli during an inhibition task and temporarily storing and manipulating information during a working memory task, both of which are particularly challenging for older adults (Baddeley, 1986; Hsieh et al., 2016). Previous work from our group has examined the differences in cognitive demand between simple reaction time, neural inhibition, and working memory tasks (Fraser et al., 2016; Potvin‐Desrochers et al., 2017; St‐Amant et al., 2020). Based on these findings, the present study will manipulate cognitive demand across these different executive function domains to determine the effects of cognitive task difficulty on neural activation and performance.

The purpose of this study was to examine how older adults mitigate the demands of dual‐tasking through changes in brain activation and behavior. The first aim was to determine the changes in cerebral oxygenation (∆HbO2 and ∆HbR) using fNIRS and performance (gait speed, cognitive response time, and accuracy) in single versus dual tasks and across four levels of cognitive task difficulty. Greater cerebral oxygenation changes were expected during the dual tasks in comparison with single tasks, and these changes were expected to increase with each successive difficulty level. Performance was expected to decrease between single and dual tasks with the most significant change occurring during the working memory tasks. The second aim was to correlate cerebral oxygenation and behavior to determine whether increased brain activation would be associated with poorer performance during the dual tasks. Understanding neural and behavioral changes in healthy older adults may help reveal whether declines are only associated with specific executive function domains.

## 2 METHODS

### 2.1 Participants

Twenty healthy older adults (*M* = 71.8 years, *SD* = 6.4 years, 10 females) were recruited from community centers across Ottawa, Canada. An a priori power analysis (power = 0.8, *f* = 0.25, α = 0.05) indicated that this sample size would be sufficient to detect significant interactions. Participant eligibility was determined using a phone screening (Table 1) whereby participants were included if they were right‐handed according to the Edinburgh Handedness Inventory (Oldfield, 1971) and did not have a diagnosed hearing impairment or hearing aid. Participants had to also be comfortable walking 15 meters without assistance and without neuromuscular or physical complaints that could affect walking (i.e., severe arthritis, Parkinson's disease, multiple sclerosis, broken bones). Cognitive status was determined using the Montreal Cognitive Assessment (MoCA) where participants were required to score ≥ 26 to ensure that they were cognitively healthy (Nasreddine et al., 2005). This study was ethically approved by the University of Ottawa Research Ethics Board (H08‐16–06), and all participants provided written informed consent before participating in the study.

**TABLE 1:** Summary of participant characteristics from the phone screening (Mean ± *SD*)

Characteristic	*n* = 20
Age (years)	71.8 ± 6.4
Gender	
Male	10
Female	10
Education (years)	17.0 ± 2.4
No. of medications	1.15 ± 1.0
No. of falls while walking	0.15 ± 0.37
No. of participants who exercise more than 2x/week	19

### 2.2 FNIRS equipment

**FIGURE 1:**
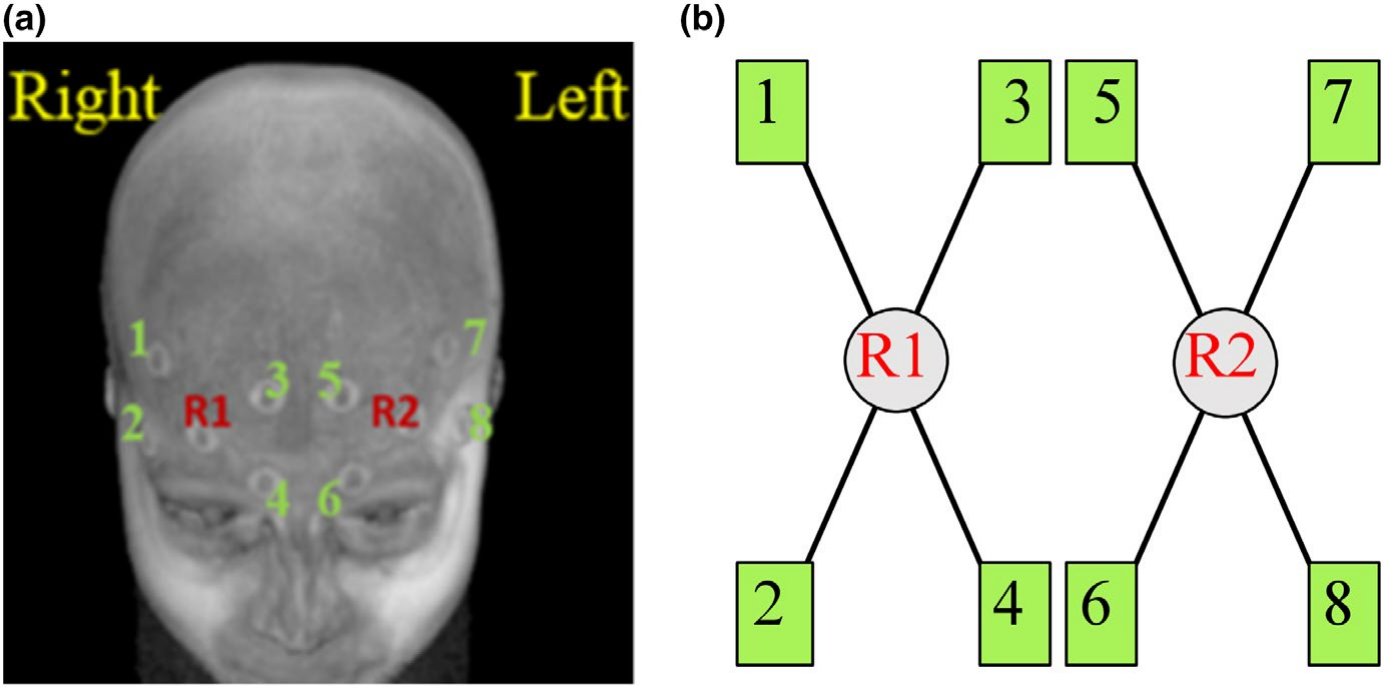
a) Localization of fNIRS optodes across the PFC. b) Optode template for the OctaMon fNIRS device that includes eight infrared light sources (1–8) and two detectors (R1 and R2)

Participants were fitted with a wearable OctaMon fNIRS device (OctaMon, Artinis, The Netherlands) to measure prefrontal ΔHbO2 and ΔHbR. The distance between the nasion and inion was measured for each participant to ensure the fNIRS device was placed along the PFC according to the modified International EEG 10–20 system (Herwig et al., 2003). The OctaMon uses continuous‐wave near‐infrared spectroscopy, which measures near‐infrared light absorption at two distinct wavelengths (760 and 850 nm). This device also uses eight light emitting diode (LED) channels and two detectors with an interoptode distance of 35 mm (Figure 1).

### 2.3 Experimental protocol

**FIGURE 2:**
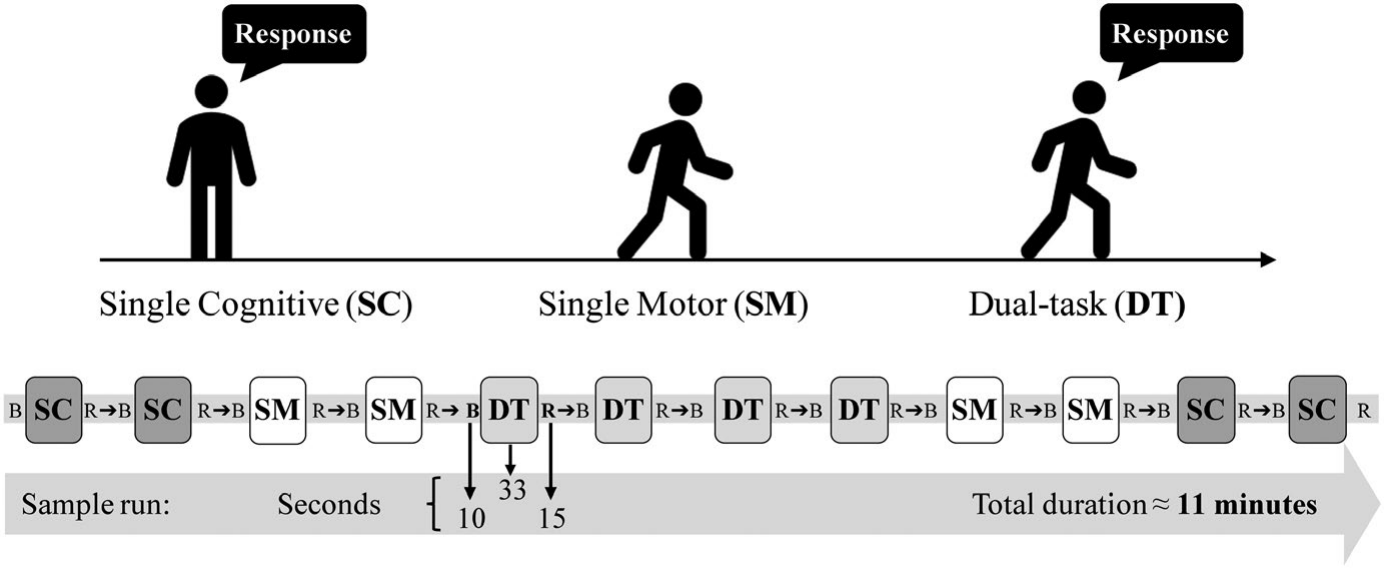
Description of a sample run including single cognitive (SC; responding to the cognitive task), single motor (SM; normal walking), and dual‐task (DT; walking with a cognitive task) blocks. Each 33 s block is preceded by a 10 s baseline and followed by a 15 s rest period. The approximate duration of a run is 11 min and is repeated for each cognitive task difficulty level

Participants were presented with four runs in a randomized order each evaluating one of four levels of cognitive demands. A run was comprised of 12 counterbalanced blocks with an equal number of single cognitive (SC), single motor (SM), and dual‐task (DT) blocks (Figure 2). In the SC condition, participants performed the cognitive task while standing and staring straight ahead at a target. The SM block had participants walk without a cognitive task at their self‐selected pace along a 10 m walkway. During the DT condition, participants were asked to perform both the cognitive and motor task simultaneously and were instructed to pay equal attention to both tasks. To gain a better understanding of the subjective emphasis dedicated to the dual tasks, participants were asked to report how much attention (out of a possible 100%) they attributed to the cognitive and motor task following the DT blocks. Each 33 s block was preceded by a 10 s baseline of quiet standing and was followed by a 15 s rest period to allow the hemodynamic response to revert to the baseline in between blocks (Herold et al., 2017). Throughout the experiment, participants were given breaks as needed and upon request.

### 2.4 Cognitive task difficulty levels

E‐Prime 2.0 software (Psychology Software Tools, Pittsburgh, PA) was used to create and present different sequences of auditory stimuli. The experimenter delivered all instructions to the participants using a microphone which could be heard through wireless headphones worn by the participant. Four cognitive‐auditory tasks: simple reaction time (SRT), go/no‐go (GNG), n‐back (NBK), and double number sequence (DNS), were chosen from previous work in our labs, to represent processing speed (SRT), neural inhibition (GNG), and working memory tasks (NBK and DNS) (Fraser et al., 2016; St‐Amant et al., 2020). During a short practice session, participants familiarized themselves with the cognitive tasks until they were able to correctly respond to 70% of the SC stimuli. The SRT task represented the simplest cognitive demand and had participants respond to a random sequence of beeps (2,850 Hz at 99 dB) by saying the word “top” as quickly as possible following each stimulus. GNG was the medium level task and had participants listen to both high‐ (2,850 Hz at 99 dB) and low‐pitched (970 Hz at 95 dB) beeps but only respond “top” to the high‐pitched beeps. The next level task was the NBK and had participants listen to a continuous sequence of single‐digit numbers (1–9) and respond with the number they heard two numbers back. Lastly, the DNS task represented the highest cognitive demand and had participants listen to a sequence of three‐digit numbers. At the end of the block, they reported the total number of times they heard two target digits within the entire sequence (Richer et al., 2017). Two working memory tasks (NBK and DNS) were chosen because working memory is the executive domain known to be most affected by cognitive aging (Baddeley, 1986).

### 2.5 Behavioral measures

Three behavioral measures were chosen to evaluate performance differences between single and dual tasks as well as across cognitive task difficulty. The first measure, gait speed (m/s), was calculated by dividing the distance the participants walked by the fixed duration of the block. Response times (s) were recorded using a voice recorder and imported into Audacity (version 2.3.1) to measure the time from stimulus onset until the participant's response. Response times were recorded during the SRT, GNG, and NBK difficulty levels. No response time was measured during the DNS condition because it is a nonverbal working memory task that has participants withhold their response until the end of the block. Finally, experimenters calculated accuracy scores (% correct) for correct responses to the cognitive tasks. In the SRT difficulty level, correct responses were recorded when the participant responded to a beep by saying the word “top” while incorrect responses were noted when the participant did not respond to a beep. Correct responses in the GNG condition were calculated when the participant correctly responded to the high‐ rather than the low‐pitched beep. Errors were noted when participants either missed the high beep or responded to the low beep. During the NBK, correct responses involved participants correctly responding with the number they heard two numbers back. Errors were given when participants responded with the incorrect number or did not respond at all. Finally, correct responses in the DNS were calculated based on the participant's final tally of each target digit compared to the total possible correct responses.

### 2.6 Test battery

Participants were asked to complete a battery of neuropsychological and physical tests. The purpose of these tests was to ensure good cognitive and physical function, low fear of falling, and no depression which may influence study outcomes. The neuropsychological tests included the Montreal Cognitive Assessment (MoCA) (Nasreddine et al., 2005), Digit Forward and Backward (Wechsler, 1981), Digit Symbol Substitution Test (Wechsler, 1981), and Trail Making Test (TMT) Part A and B (Strauss et al., 2006). The MoCA is a screening tool used to assess cognitive impairment. Individuals who score ≥ 26 out of 30 reflect healthy cognition (Nasreddine et al., 2005). Digit Forward and Backward are used to assess working memory, and points were awarded for correctly repeating a growing list of numbers in either the forward or reverse direction. A higher digit span indicates a better memory span. The Digit Symbol Substitution Test measures processing speed as individuals fill‐in as many symbols as possible within 90 s based on a key provided at the top of the worksheet. A higher score indicates more efficient cognitive processing. The Trail Making Tests are timed tests (s) used to measure task switching and executive functioning. It is divided into two parts whereby Part A has participants draw lines connecting 25 ascending numbers while Part B has participants draw lines alternating between ascending numbers and letters. A shorter time to complete these tests indicates better performance. Furthermore, physical status and fear of falling were assessed using the Short Physical Performance Battery (SPPB) and the Falls Efficacy Scale‐International (FES‐I), respectively. The SPPB measures lower extremity functioning in older adults and is scored out of 12, where 12 is equivalent to no deficits in functioning (Guralnik et al., 1994). FES‐I uses a 4‐point Likert scale to assess an individual's fear of falling (Delbaere et al., 2010). It is scored out of 64 whereby a higher score indicates a greater fear of falling. The Geriatric Depression Scale (GDS) was also used to assess depression in older adults as it is known to have effects on the PFC (Yesavage & Sheikh, 1986). It is scored out of 30, and a lower score within the range of 0–9 indicates no depression.

### 2.7 Data processing of fNIRS signal

Neural data were collected in Oxysoft (Artinis, The Netherlands, version 3.0.97.1) and sampled at a frequency of 10 Hz. After visually inspecting the signal quality, the Modified Beer–Lambert law was applied to the raw HbO2 and HbR intensities using a differential pathlength factor set to 6.61 for all older adults (Scholkmann & Wolf, 2013). The concentrations were then preprocessed offline using a custom MATLAB (R2018a) script. The script eliminated motion artifacts by removing outliers that were 2.5 *SD* from the mean and replaced them with a zero value. Additionally, in line with the literature, a Butterworth band‐pass filter set between 0.01 and 0.14 Hz was used to reduce physiological noise (heartbeat and breathing) within the signal (Holtzer et al., 2011; Mirelman et al., 2017; Verghese et al., 2017). An average ∆HbO2 and ∆HbR value were then calculated in µM for each task (SC, SM, DT) and each difficulty level (SRT, GNG, NBK, DNS) from the changes in signal between the baseline and active conditions.

### 2.8 Statistical analyses

Differences in cerebral oxygenation (∆HbO2 and ∆HbR) were assessed using 2x4 repeated measures ANOVAs whereby task (SC/SM versus. DT) and difficulty (SRT, GNG, NBK, DNS) main effects and interactions were tested.

Assessments of behavioral response time were tested with a 2x3 repeated measures ANOVA to measure the interaction between task (SC, DT) and difficulty (SRT, GNG, NBK). Note that the DNS task had participants respond at the end of the block; therefore, no response time was calculated. Significant differences in gait speed and accuracy were evaluated with 2x4 repeated measures ANOVAs to measure the interaction between task (SC/SM versus. DT) and difficulty (SRT, GNG, NBK, DNS).

A one‐way ANOVA was conducted on the subjective emphasis responses to test whether there were significant differences between how much attention the participants dedicated to walking versus the cognitive tasks across each difficulty level (SRT, GNG, NBK, DNS).

For all repeated measures ANOVAs, if Mauchly's Test of Sphericity was violated, a Greenhouse–Geisser *p*‐value was reported. In addition, Bonferroni post hoc analysis was used to determine the location of significance where statistical significance was set at *p* < .05. Means (*M*) and standard deviations (*SD*) are reported in the results, and when a distinction between difficulty levels is needed, the difficulty level is identified in subscript (i.e., *M*_SRT_ = Mean value for SRT difficulty level). Means and standard deviations were calculated for all participant demographics and neuropsychological assessments.

No significant differences were observed in terms of cerebral oxygenation between channels or hemispheres (*p*‐values > .05). Therefore, brain activation was analyzed across the whole PFC by averaging the concentration output from each channel. In addition, we verified if there were significant changes in cerebral oxygenation within task (e.g., the four SM blocks in SRT) and there were no significant differences (*p*‐values > .90). As such, an average of each task type was calculated for analyses.

A Pearson correlation was used to examine the relationship between cerebral oxygenation (ΔHbO2 and ΔHbR) and performance (gait speed, response time, and accuracy) during the dual tasks.

## 3 RESULTS

### 3.1 Neural: Changes in cerebral oxygenation

**FIGURE 3:**
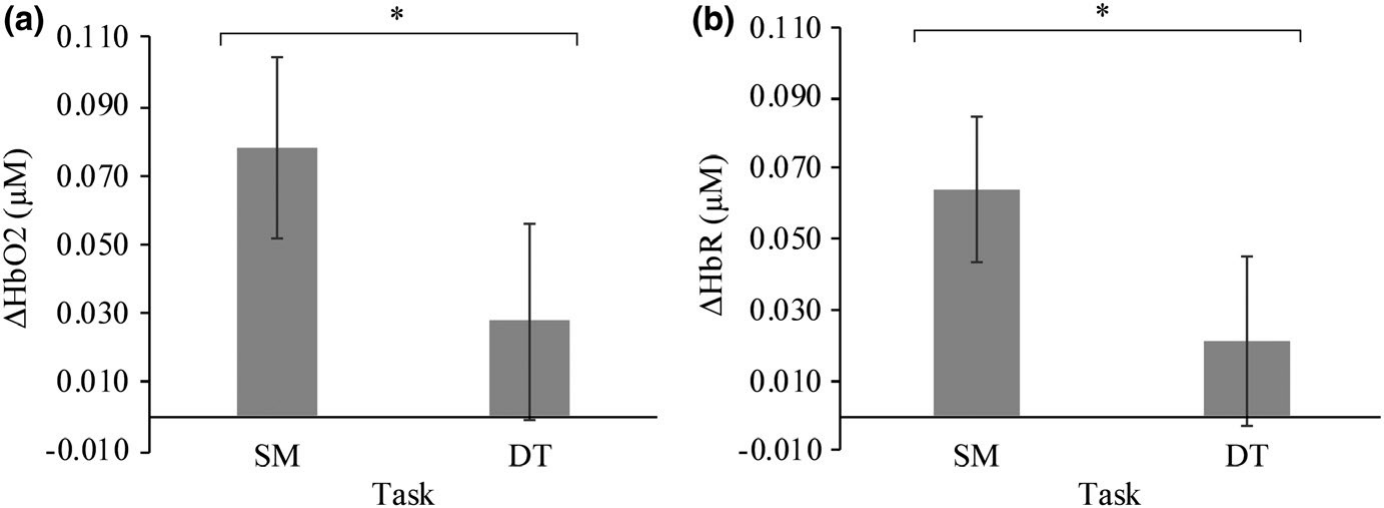
a) Change in prefrontal cerebral oxygenation (∆HbO2) *F* (1,19) = 4.5, *p* = .047, *η^2^* = 0.191 between single motor (SM) and dual task (DT). There was a significant decrease in PFC activation during the dual versus single task (*p* = .047). b) Change in prefrontal cerebral deoxygenation (∆HbR) *F* (1, 19) = 4.8, *p* = .040, *η^2^* = 0.203 between single motor (SM) and dual‐task (DT) blocks. Cerebral deoxygenation in the PFC significantly decreased between single and dual tasks (*p* = .040). (*) indicates significance *p* < .05. Error bars represent standard error of the mean

**FIGURE 4:**
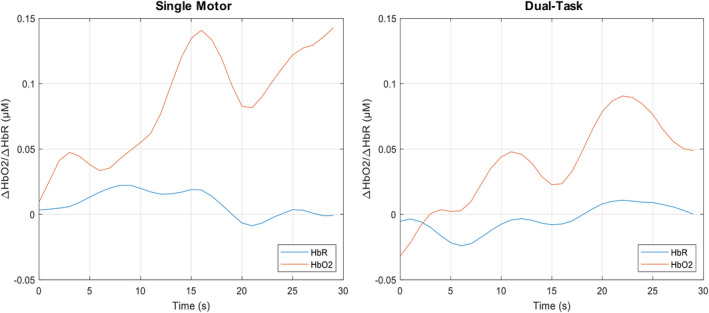
Mean hemodynamic response across all participants in the single motor and dual‐task blocks. Neural findings demonstrated a main effect of task such that oxygenated hemoglobin (HbO2; *F* (1,19) = 4.5, *p* = .047, *η^2^* = 0.19) and deoxygenated hemoglobin (HbR; *F* (1, 19) = 4.8, *p* = .040, *η^2^* = 0.203) significantly decreased between single motor and dual tasks. The blue and red lines represent HbR and HbO2, respectively

A significant main effect of task on ∆HbO2 was observed *F* (1,19) = 4.5, *p* = .047, *η^2^* = 0.191 (Figure 3a). A post hoc analysis revealed that ∆HbO2 significantly decreased (*p* = .047) from SM (*M* = 0.078 µM, *SD* = 0.026 µM) to DT (*M* = 0.028 µM, *SD* = 0.029 µM). There was also a main effect of task on ∆HbR *F* (1, 19) = 4.8, *p* = .040, *η^2^* = 0.203 (Figure 3b). The post hoc analysis indicated that ∆HbR significantly decreased (*p* = .040) from SM (*M* = 0.064 µM, *SD* = 0.021 µM) to DT (*M* = 0.021 µM, *SD* = 0.024 µM). A normal distribution of the ∆HbO2 and ∆HbR signals over the course of SM and DT blocks has been depicted in Figure 4. There were no significant interactions between task (SC, DT) and difficulty (SRT, GNG, NBK, DNS) for ∆HbO2 (*p = *.400) and ∆HbR (*p = *.412) or main effects of task (∆HbO2; *p = *.200, HbR; *p = *.169) and difficulty (∆HbO2; *p = *.414, ∆HbR; *p = *.476).

### 3.2 Behavioral: Changes in response time, accuracy, and gait speed

**FIGURE 5:**
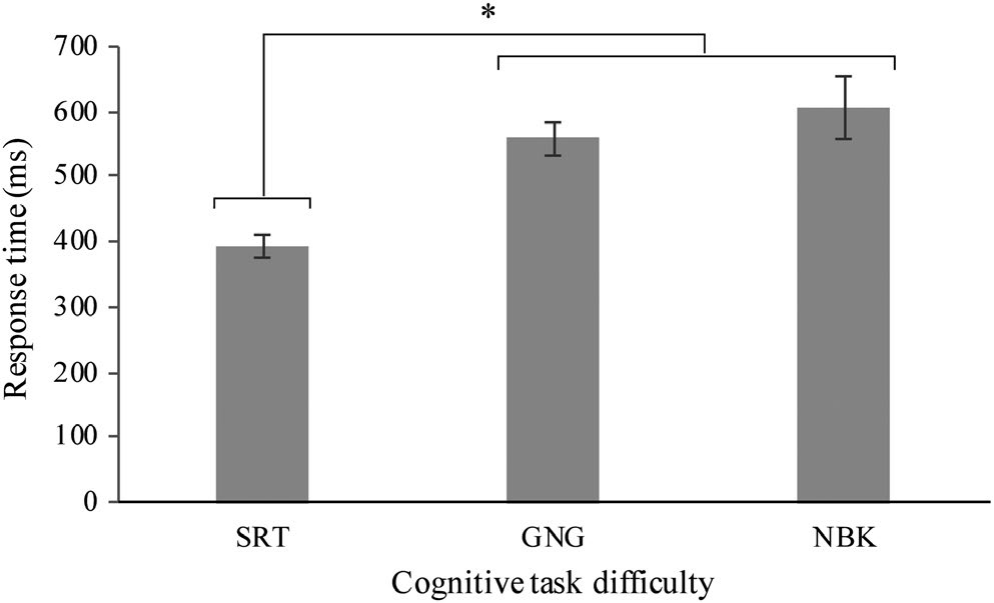
Mean response time (ms) changes between cognitive task difficulty levels (SRT), go/no‐go (GNG) and n‐back (NBK) SRT *F* (2, 38) = 16.0, *p* < .001, *η^2^* = 0.456. Response times in the GNG and NBK were significantly slower than the SRT (*p* < .001). (*) indicates significance *p* < .001. Error bars represent standard error of the mean

Response time (ms) increased across increasing levels of difficulty whereby SRT < GNG <NBK (*M*_SRT_ = 394 ms, *SD*_SRT_ = 86.3 ms; *M*_GNG_ = 559 ms, *SD*_GNG_ = 116 ms; *M*_NBK_ = 605 ms, *SD*_NBK_ = 206 ms). This was demonstrated by a main effect of difficulty on response time *F* (2, 38) = 16.0, *p* < .001, *η^2^* = 0.456 (Figure 5). Post hoc analysis indicated that SRT response times were significantly faster than the GNG and NBK conditions (*p* < .001).

**FIGURE 6:**
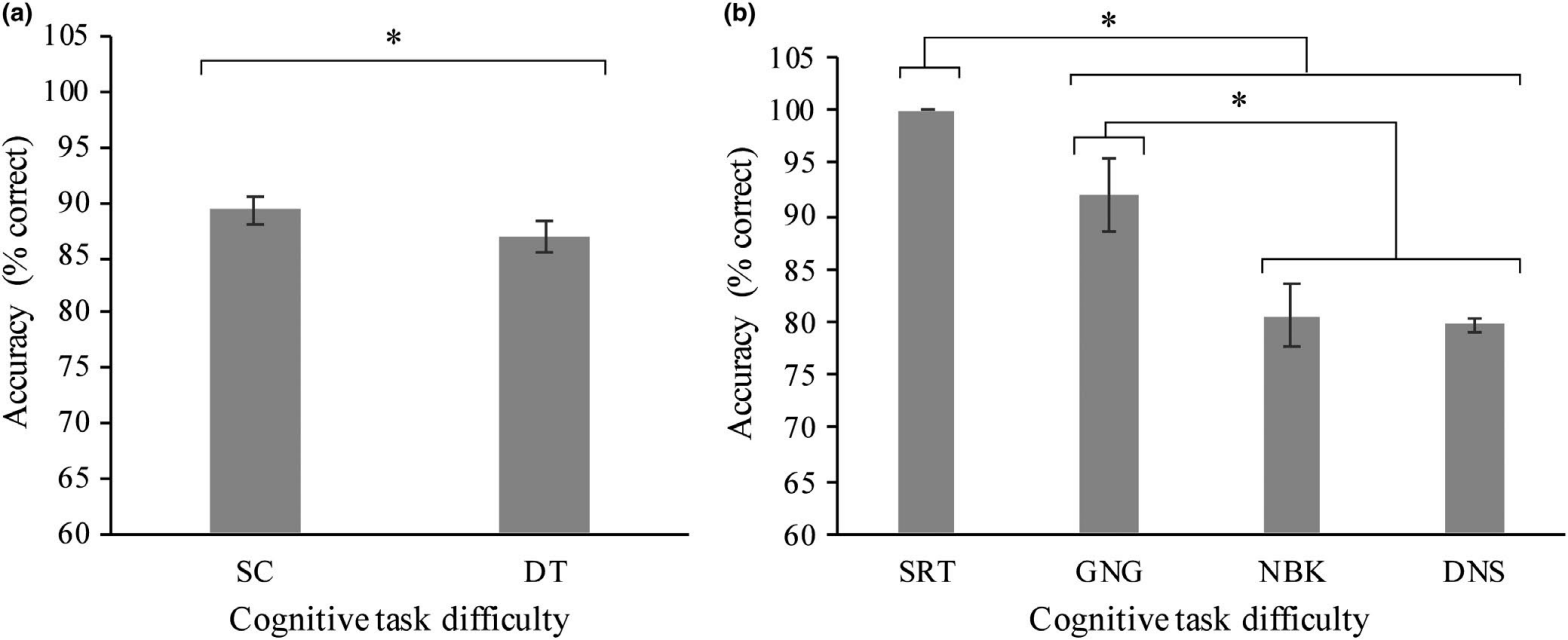
a) Mean accuracy decrease between single cognitive (SC) and dual‐task (DT) *F* (1, 19) = 5.7, *p* = .028, *η* ^2^ = 0.230. SC was significantly more accurate than DT (*p* < .001). b) Mean decrease in accuracy (% correct) across cognitive task difficulty levels including simple reaction time (SRT), go/no‐go (GNG), n‐back (NBK), and double number sequence (DNS) *F* (3, 57) = 16.2, *p* < .001, *η* ^2^ = 0.460. Participants were significantly more accurate during the SRT than the GNG (*p* = .038), NBK (*p* < .001), and DNS (*p* < .001), and in the GNG compared to NBK (*p* = .042) and DNS (*p* = .002). (*) indicates significance *p* < .05. Error bars represent standard error of the mean

Analyses revealed a main effect of task on accuracy *F* (1, 19) = 5.7, *p* = .028, *η^2^* = 0.230 (Figure 6a). Post hoc tests revealed that SC (*M* = 89.3%, *SD* = 13.5%) was significantly more accurate than DT (*M* = 86.9%, *SD* = 14.4%, *p* < .001). There was also a main effect of difficulty *F* (3, 57) = 16.2, *p* < .001, *η^2^* = 0.460, whereby accuracy decreased as the cognitive tasks became more difficult (*M*_SRT_ = 100%, *SD*_SRT_ = 0.0%; *M*_GNG_ = 92.0%, *SD*_GNG_ = 17.0%; *M*_NBK_ = 80.6%, *SD*_NBK_ = 15.0%; *M*_DNS_ = 79.7%, *SD*_DNS_ = 4.83%) (Figure 6b). Post hoc tests revealed that responses in SRT were significantly more accurate than GNG (*p* = .038), NBK (*p* < .001) and DNS (*p* < .001). In addition, GNG was more accurate than NBK (*p* = .042) and DNS (*p* = .002); however, NBK and DNS were not significantly different (*p = *.740).

**FIGURE 7:**
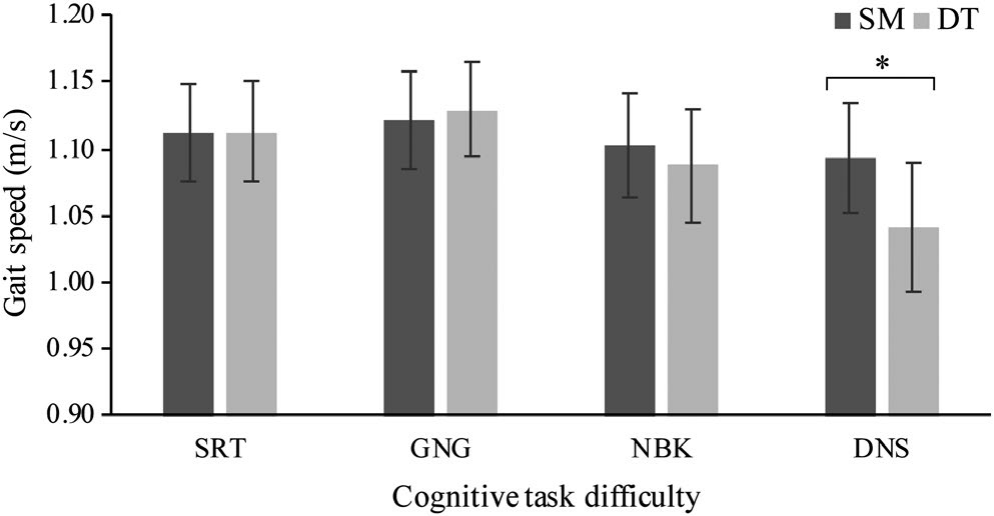
Mean gait speed changes (m/s) between single motor (SM) and dual‐task (DT) blocks and across cognitive task difficulty levels *F* (3, 57) = 2.2, *p* = .014, *η^2^* = 0.169. Cognitive tasks include simple reaction time (SRT), go/no‐go (GNG), n‐back (NBK), and double number sequence (DNS). Mean gait speed was significantly slower between the DNS single and dual task (*p* = .003). (*) indicates significance *p* < .05. Error bars represent standard error of the mean

An interaction effect between task (SM, DT) and difficulty (SRT, GNG, NBK, DNS) was observed for gait speed, *F* (3, 57) = 2.2, *p* = .014, *η^2^* = 0.169 (Figure 7). Post hoc analyses indicated that during the most difficult cognitive task, the DNS, there was a significant decrease (*p* = .003) in gait speed between SM (*M* = 1.11 m/s, *SD* = 0.38 m/s) and DT (*M* = 1.09 m/s, *SD* = 0.38 m/s). There were no significant differences between single‐ and dual‐task gait speed during the SRT (*p* = .772), GNG (*p* = .706) and NBK (*p* = .379) cognitive tasks.

The ANOVA on subjective emphasis revealed a significant decrease in the attention dedicated to walking across cognitive task difficulty *F* (3, 57) = 14.8, *p* < .001, *η^2^* = 0.438. The participants reported focusing less on walking with each successive difficulty level (*M*_SRT_ = 39.1%, *SD*_SRT_ = 18.0%; *M*_GNG_ = 31.4%, *SD*_GNG_ = 15.2%; *M*_NBK_ = 22.6%, *SD*_NBK_ = 17.4%; *M*_DNS_ = 18.9%, *SD*_DNS_ = 19.8%). Post hoc analyses revealed that participants focused significantly less on walking during the NBK (*p* < .001) and DNS (*p* = .001) compared to the SRT and significantly less in the NBK (*p* = .038) and DNS (*p* = .017) compared to the GNG. There were no significant differences between the SRT and GNG (*p* = .056) and the NBK and DNS (*p* = 1.000).

### 3.3 Correlation between cerebral oxygenation and behavior

There were no significant correlations between cerebral oxygenation (∆HbO2; *p*‐values > 0.081) and deoxygenation (∆HbR; *p*‐values > .068) and behavior (response time, response accuracy, gait speed) during the dual tasks.

## 4 DISCUSSION

The current study applied fNIRS imaging to assess whether older adults demonstrated changes in prefrontal cerebral oxygenation and behavior while walking with cognitive tasks of increasing difficulty. The aims of this study were twofold. First, to analyze neural and behavioral measures to better understand neural compensation mechanisms during dual tasks of different difficulty levels. Second, to determine whether there was a correlation between neural and behavioral outcomes such that increases PFC activation may be associated with better performance, or vice versa, in older adults. In doing so, this may reveal how older adults mitigate their attention capacity through prefrontal executive involvement or adopt compensatory neural strategies to meet the demands of difficult dual tasks.

### 4.1 Neural

According to our initial hypothesis, ∆HbO2 was expected to increase from single to dual tasks based on the principles of STAC‐r (Reuter‐Lorenz & Park, 2014). This prediction was based on the neuroimaging literature which suggests that older adults exhibit more widespread and bilateral activation in the PFC during dual versus single tasks and, therefore, greater dependency on executive control compared to younger adults (Fraser et al., 2016; Holtzer et al., 2011). Contrary to this expectation, this study demonstrated a significant decrease in ∆HbO2 and ∆HbR between walking alone (i.e., single task) and walking with a cognitive task (i.e., dual task). These findings are in line with several reports that observed a decrease of prefrontal cerebral oxygenation and an alternative strategy to mitigate the demands of dual‐task walking (Beurskens et al., 2014; Pelicioni et al., 2019). One possibility is an automatic locomotor control strategy which would be beneficial in dual‐task situations to minimize interference with other controlled processes (Clark, 2015; Schneider & Shiffrin, 1977). The PFC’s contributions to walking include managing the attentional demands and motor planning associated with safe and efficient displacement (Clark, 2015; Yogev et al., 2008). However, executive resources are limited and may be reorganized depending on task demands. Studies have shown that decreased PFC activation is associated with automatically controlled tasks and walking, in particular, is amenable to automation because it is well learned (Dietrich & Audiffren, 2011; Wu et al., 2004). Therefore, increased prefrontal activation may only be observed in individuals who show a loss of automaticity such as in people with neurological disorders or frail older adults (Beurskens & Bock, 2012; Holtzer et al., 2016; Maidan et al., 2016; Woollacott & Shumway‐Cook, 2002). Based on the data presented in Table 2, the older adults in this study demonstrated high scores in cognitive function, walk speed (i.e., >1 m/s) and no frailty, among other factors, which are typically associated with decreased executive functioning. These measures suggest that our participant group was high functioning and could rely on an automatic locomotor strategy to free up cognitive resources in the PFC.

**TABLE 2:** Mean neuropsychological and health status test scores (Mean ± *SD*)

Test	*n* = 20
MoCA (/30)	27.2 ± 1.2
Digit Forward (score/16)	10.7 ± 1.6
Digit Backward (score/14)	7.3 ± 1.9
Digit Symbol Substitution Test (# of symbols /93)	45.0 ± 9.7
TMT A (s)	37.9 ± 12.7
TMT B (s)	83.3 ± 25.6
SPPB (/12)	11.1 ± 1.6
FES‐I (/64)	20.8 ± 3.6
GDS (/30)	3.2 ± 3.0

Participants were also asked to subjectively rate how much attention they paid toward the cognitive versus walking task. Their responses reflected an automatic control strategy in that they reported focusing <39% on walking during all the cognitive tasks. The cognitive tasks may have also served as an external focus which has been known to facilitate automatic processing (Bernstein, 1967; Poldrack, 2005). This has been outlined in the “constrained action hypothesis” which suggest that focusing on the outcome of a movement (i.e., external focus), rather than the movement itself (i.e., internal focus), minimizes interference with other consciously controlled tasks (Wulf, 2013; Wulf et al., 2001). Similarly, diverting attention away from a postural task (i.e., to a cognitive task) even when cognitive demands are low may provide an external focus to improve motor performance (Huxhold et al., 2006). As such, compared to walking alone, responding to the various stimuli during the dual tasks may have helped draw attention away from walking and allowed for greater stability without greater recruitment of the PFC. Conversely, in the absence of a cognitive task, attention could be drawn to both internal and external sources thereby engaging greater executive control.

Healthy individuals inherently shift between automatic and executive control strategies to mitigate cognitive demands (Clark, 2015; Yogev et al., 2008). However, studies have also demonstrated age‐related decreases in cerebral blood flow (CBF) to the PFC due to changes in brain structure (Bertsch et al., 2009). The reorganization of locomotor control pathways and a reduction of CBF with age may, therefore, contribute to an overall reduced availability of prefrontal oxygenation. Dietrich’s (2003) theory of hypofrontality suggests that there is a redistribution of metabolic resources from prefrontal brain regions to motor regions during tasks such as walking due to the complex integration of sensory, motor, and autonomic processes. In other words, the brain is limited by a finite supply of metabolic resources that must be strategically allocated based on the most critical demands (Dietrich, 2003). Taken together with automaticity, hypofrontality may cause a downregulation of metabolic resources in the PFC which can be redistributed to other brain regions to supplement motor control. Regions outside the PFC could not be measured within the scope of this study; however, studies have shown heightened brain activation in motor areas such as the premotor (Lu et al., 2015) and supplemental motor area (Harada et al., 2009; Lu et al., 2015; Miyai et al., 2001) during dual‐task walking. These brain regions should be further examined simultaneously with the PFC to determine whether a decrease in prefrontal cerebral oxygenation from single to dual task corresponds with changes in motor regions when walking more automatically.

We must also acknowledge certain study parameters including the (a) cognitive and (b) motor tasks that differentiate this study from others in the literature. (a) Cognitive tasks: Verbal fluency (Hawkins et al., 2018; Holtzer et al., 2015; Verghese et al., 2017) and counting backwards (Al‐Yahya et al., 2016; Mirelman et al., 2017) are the most commonly used tasks in dual‐task studies that demonstrate increased or no change in cerebral oxygenation between single and dual tasks (Pelicioni et al., 2019). Our study used processing speed, neural inhibition, and working memory tasks which continuously prompted responses and engaged participants based on a random sequence of stimuli. This differs from verbal fluency and counting tasks in that participants were not provided with a starting cue (i.e., a letter or number) after which they could respond at their own pace. The external focus of the cognitive tasks and unpredictable pattern of stimuli may have helped recruit automatic control pathways by ensuring that the full duration of the task was attention‐demanding (Beck et al., 2018; Bernstein, 1967). (b) Motor task: Walking trajectories vary significantly across studies due to equipment and space constraints. As evidenced by studies examining obstacle negotiation, the interruption of steady state walking caused increased PFC activation and may equally impede automaticity (Hawkins et al., 2018; Holtzer et al., 2016; Maidan et al., 2016). Our study provided participants with a 10 m pathway to maximize straight‐line walking which is considerably longer than studies examining gait along electronic walkways (Hernandez et al., 2016; Holtzer et al., 2011; Mirelman et al., 2017; Verghese et al., 2017). Therefore, our walking task provided longer stretches of steady state walking and a greater opportunity to automatize gait than studies using shorter walkways.

Lastly, in addition to the ΔHbO2 decrease, there was also a decrease in ΔHbR between the single and dual tasks. ΔHbR is a reliable measure of neural activation but is less commonly reported in the literature. This is due to its low signal amplitude making significant changes between baseline and task conditions more difficult to obtain (Leff et al., 2011). The low signal amplitude also means that HbR is less likely to be contaminated with physiological artifacts and also results in a lower signal to noise ratio (Leff et al., 2011). As such, capturing a significant HbR change that mirrors the HbO2 findings further supports a decrease in brain activation between single and dual tasks.

### 4.2 Behavioral

Examining gait speed in older adults alongside behavioral measures such as response time and accuracy may offer insights into the cognitive‐motor interactions underlying dual‐task walking. Gait speed changes in older adults have been well documented in the literature such that increasing attentional demands while walking may affect walking performance (Hausdorff et al., 2008; Smith et al., 2016; Yogev et al., 2008). Findings from the present study partially support this in that gait speed decreased but only during the most difficult cognitive task. Gait speed maintenance across the first three levels of task difficulty may be explained by an automatic locomotor control strategy, as described in the neural findings. However, this strategy may not have been sufficient to mitigate the demands of the DNS dual task. As suggested in the “posture first hypothesis,” older adults subconsciously prioritize gait over cognitive performance to ensure safe ambulation (Holtzer et al., 2016; Shumway‐Cook et al., 1997; Yogev‐Seligmann et al., 2012). Slowing gait speed may, therefore, be a combination of prioritization and compensation strategies to ensure older adults can function safely under complex task demands. It is worth noting that older adults commonly decrease their gait speed <1.0 m/s during dual tasks which is also a cutoff used to identify individuals who are at a greater risk of falls (Hollman et al., 2011; Kyrdalen et al., 2019; Smith et al., 2016; Verghese et al., 2017). When the older adults in this study decreased their gait speed during the most difficult task, it still remained on average >1.0 m/s. This may further indicate the physical status of the participants which could have an impact on performance as compared to other studies in the literature (Dupuy et al., 2015; Holtzer et al., 2015).

Decreased response time and accuracy performance may also be a consequence of gait prioritization. Our findings demonstrated increased response times from the easiest to the most demanding task. More specifically, the response times in the SRT task were significantly faster than the GNG and NBK tasks. However, the GNG and NBK tasks were not significantly different from one another. This was expected in that compared to the SRT task, the GNG and NBK tasks involved more complex processing steps. For example, the simple reaction time task required a response after each stimulus whereas the GNG task forced the older adults to first discriminate between a “go” and “no‐go” stimulus before responding (Hsieh et al., 2016). Similarly, the NBK working memory task involved maintaining and updating information before responding to the stimuli (Al‐Yahya et al., 2011). Based on these findings, more complex processing steps require more processing capacity. This was evident during the more difficult tasks as the older adults slowed their response times significantly during the inhibition and the working memory tasks compared to the SRT task. Further, the older adults responded less accurately as the difficulty level increased. However, there were no differences between the working memory tasks. These findings support our difficulty manipulation such that participants were most accurate during the processing speed task and least accurate during the working memory tasks.

In line with the literature, increasing task difficulty was expected to result in lower accuracy (Fraser et al., 2016; Srygley et al., 2009; Vermeij et al., 2012). Interestingly, participants maintained their accuracy >80% throughout all the dual tasks. This suggests that a high level of performance is achievable with increasing cognitive demands when cognitive resources are allocated effectively. However, participants were less accurate during the dual‐ versus single tasks. This has been demonstrated in the literature whereby participants make more errors during dual tasks due to the competing demands of performing two tasks simultaneously (Brustio et al., 2017; Srygley et al., 2009).

### 4.3 Correlation between cerebral oxygenation and behavior

There were no significant correlations between the changes in cerebral oxygenation and behavioral performance. More specifically, the changes in cerebral oxygenation across task and difficulty were not associated with gait speed, response time or accuracy performance. This could be due to the small sample of older adults in this study. However, interpreting neural and behavioral findings together revealed that the redistribution of metabolic resources in the PFC may have contributed to insignificant differences in gait speed across the first three levels of task difficulty. The same cannot be said for response time and accuracy performance in which decreased cerebral oxygenation in the PFC did not result in behavioral gains. Future studies should examine automaticity and neural efficiency across task difficulty in regions outside the PFC as certain regions of interest may increase or decrease activity with the maintenance and decline of different performance measures. Follow‐up studies should be conducted to determine how this impacts cognition in the long term. This may equally reveal whether individuals exhibiting decrements in behavior due to neural inefficiency may be at a greater risk of cognitive decline.

### 4.4 Limitations

Gait parameters were only quantified using gait speed. Gait speed is commonly used in the literature because it is easily collected in clinical settings, requires minimal equipment and is a good indicator of motor performance in older adults (Holtzer et al., 2015). However, other measures that capture gait variability including stride length or stride time could complement gait speed measures and may provide greater insight into subtle changes in dual‐task performances, different age groups, and different clinical populations. In addition, the choice of fNIRS device limited our data acquisition to the PFC. This device facilitated setup and caused minimal discomfort for the participants; however, we can only speculate as to which other brain regions were involved in dual‐tasking and the potential executive–automatic processing shift in walking with increasing difficulty. Despite this, fNIRS has a high temporal resolution compared to other techniques such as fMRI and is a reliable tool for measuring cerebral oxygenation in the PFC (Pinti et al., 2018).

## 5 CONCLUSION

Executive functions are known to decline with age and can significantly affect the way older adults divide their attention between two simultaneous tasks. Many older adults adapt to these changes by using compensatory neural strategies to accomplish tasks exceeding their cognitive capacity. The neural findings of this study suggest that an automatic locomotor control strategy can decrease the recruitment of executive resources in the PFC during dual versus single tasks. Behaviorally, this allowed for gait speed maintenance until the most difficult working memory task after which older adults slowed down to mitigate the cognitive task demands. Consequently, prioritizing gait led to slower response times and worse response accuracy across task difficulty.

Findings from this study helped reveal the PFC’s role in allocating cognitive resources during processing speed, neural inhibition, and working memory tasks while walking. Evaluating automatic processes has clinical applications in that a loss of automaticity may be associated with frailty. Similarly, dual‐task performance declines measured across different task domains may be used to inform interventions that delay or prevent subsequent decline. Future studies can develop an even better understanding of this relationship as neuroimaging becomes more portable, more extensive (i.e., covering the entire brain), and adaptable to different environments. In particular, assessing dual‐task walking in real‐life situations such as crossing the street while talking on the phone may generate more novel approaches to understanding executive and controlled processes within the scope of cognitive aging.

## AUTHOR CONTRIBUTIONS

SF and YL contributed to the study design and implementation. TS, NP, LM, and GS assisted with data collection. DT created the MATLAB script. TS and SF contributed to the analysis and interpretation of the findings. TS drafted the original manuscript. All authors reviewed and approved the final version of the manuscript.

## Data Availability

Data available on request due to privacy/ethical restrictions.

## References

[brb32021-bib-0001] Al‐Yahya, E. , Dawes, H. , Smith, L. , Dennis, A. , Howells, K. , & Cockburn, J. (2011). Cognitive motor interference while walking: A systematic review and meta‐analysis. Neuroscience & Biobehavioral Reviews, 35(3), 715–728. 10.1016/j.neubiorev.2010.08.008 20833198

[brb32021-bib-0002] Al‐Yahya, E. , Johansen‐Berg, H. , Kischka, U. , Zarei, M. , Cockburn, J. , & Dawes, H. (2016). Prefrontal cortex activation while walking under dual‐task conditions in stroke: A multimodal imaging study. Neurorehabilitation and Neural Repair, 30(6), 591–599. 10.1177/1545968315613864 26493732 PMC5404717

[brb32021-bib-0003] Baddeley, A. (1986). Working memory. Oxford University Press.

[brb32021-bib-0004] Beck, E. , Intzandt, B. , & Almeida, Q. J. (2018). Can dual task walking improve in Parkinson’s disease after external focus of attention exercise? A single blind randomized controlled trial. Neurorehabilitation and Neural Repair, 32(1), 18–33. 10.1177/1545968317746782 29262749

[brb32021-bib-0005] Bernstein, N. (1967). The co‐ordination and regulation of movements. New York, Pergamon Press.

[brb32021-bib-0006] Bertsch, K. , Hagemann, D. , Hermes, M. , Walter, C. , Khan, R. , & Naumann, E. (2009). Resting cerebral blood flow, attention, and aging. Brain Research, 1267, 77–88. 10.1016/j.brainres.2009.02.053 19272361

[brb32021-bib-0007] Beurskens, R. , & Bock, O. (2012). Age‐related deficits of dual‐task walking: A review. Neural Plasticity, 2012, 1–9. 10.1155/2012/131608 PMC3403123 22848845

[brb32021-bib-0008] Beurskens, R. , Helmich, I. , Rein, R. , & Bock, O. (2014). Age‐related changes in prefrontal activity during walking in dual‐task situations: A fNIRS study. International Journal of Psychophysiology, 92(3), 122–128. 10.1016/j.ijpsycho.2014.03.005 24681355

[brb32021-bib-0009] Brustio, P. , Magistro, D. , Zecca, M. , Rabaglietti, E. , & Liubicich, M. (2017). Age‐related decrements in dual‐task performance: Comparison of different mobility and cognitive tasks. A cross sectional study. PLoS One, 12(7), e0181698. 10.1371/journal.pone.0181698 28732080 PMC5521845

[brb32021-bib-0010] Cabeza, R. , Albert, M. , Belleville, S. , Craik, F. , Duarte, A. , Grady, C. , Lindenberger, U. , Nyberg, L. , Park, D. C. , Reuter‐Lorenz, P. , Rugg, M. , Steffener, J. , & Rajah, M. N. (2018). Maintenance, reserve and compensation: The cognitive neuroscience of healthy ageing. Nature Reviews Neuroscience, 19(11), 701–710. 10.1038/s41583-018-0068-2 PMC6472256 30305711

[brb32021-bib-0011] Clark, D. (2015). Automaticity of walking: Functional significance, mechanisms, measurement and rehabilitation strategies. Frontiers in Human Neuroscience, 9, 10.3389/fnhum.2015.00246 PMC4419715 25999838

[brb32021-bib-0012] Delbaere, K. , Close, J. , Mikolaizak, A. , Sachdev, P. , Brodaty, H. , & Lord, S. (2010). The falls efficacy scale international (FES‐I). A comprehensive longitudinal validation study. Age and Ageing, 39(2), 210–216. 10.1093/ageing/afp225 20061508

[brb32021-bib-0013] Dietrich, A. (2003). Functional neuroanatomy of altered states of consciousness: The transient hypofrontality hypothesis. Consciousness and Cognition, 12(2), 231–256. 10.1016/S1053-8100(02)00046-6 12763007

[brb32021-bib-0014] Dietrich, A. , & Audiffren, M. (2011). The reticular‐activating hypofrontality (RAH) model of acute exercise. Neuroscience & Biobehavioral Reviews, 35(6), 1305–1325. 10.1016/j.neubiorev.2011.02.001 21315758

[brb32021-bib-0015] Dupuy, O. , Gauthier, C. , Fraser, S. , Desjardins‐Crèpeau, L. , Desjardins, M. , Mekary, S. , Lesage, F. , Hoge, R. , Pouliot, P. , & Bherer, L. (2015). Higher levels of cardiovascular fitness are associated with better executive function and prefrontal oxygenation in younger and older women. Frontiers in Human Neuroscience, 9, 10.3389/fnhum.2015.00066 PMC4332308 25741267

[brb32021-bib-0016] Fraser, S. , Dupuy, O. , Pouliot, P. , Lesage, F. , & Bherer, L. (2016). Comparable cerebral oxygenation patterns in younger and older adults during dual‐task walking with increasing load. Frontiers in Aging Neuroscience, 8, 10.3389/fnagi.2016.00240 PMC5071361 27812334

[brb32021-bib-0017] Grady, C. (2000). Functional brain imaging and age‐related changes in cognition. Biological Psychology, 54(1), 259–281. 10.1016/S0301-0511(00)00059-4 11035226

[brb32021-bib-0018] Guralnik, J. , Simonsick, E. , Ferrucci, L. , Glynn, R. , Berkman, L. , Blazer, D. , Scherr, P. , & Wallace, R. (1994). A short physical performance battery assessing lower extremity function: Association with self‐reported disability and prediction of mortality and nursing home admission. Journal of Gerontology, 49(2), M85–M94. 10.1093/geronj/49.2.M85 8126356

[brb32021-bib-0019] Harada, T. , Miyai, I. , Suzuki, M. , & Kubota, K. (2009). Gait capacity affects cortical activation patterns related to speed control in the elderly. Experimental Brain Research, 193(3), 445–454. 10.1007/s00221-008-1643-y 19030850

[brb32021-bib-0020] Hausdorff, J. , Schweiger, A. , Herman, T. , Yogev‐Seligmann, G. , & Giladi, N. (2008). Dual task decrements in gait among healthy older adults: Contributing factors. The Journals of Gerontology. Series A, Biological Sciences and Medical Sciences, 63(12), 1335–1343. 10.1093/gerona/63.12.1335 PMC3181497 19126846

[brb32021-bib-0021] Hawkins, K. , Fox, E. , Daly, J. , Rose, D. , Christou, E. A. , McGuirk, T. , Otzel, D. , Butera, K. , Chatterjee, S. , & Clark, D. (2018). Prefrontal over‐activation during walking in people with mobility deficits: Interpretation and functional implications. Human Movement Science, 59, 46–55. 10.1016/j.humov.2018.03.010 29604488 PMC5988641

[brb32021-bib-0022] Hernandez, M. , Holtzer, R. , Chaparro, G. , Jean, K. , Balto, J. , Sandroff, B. , Izzetoglu, M. , & Motl, R. (2016). Brain activation changes during locomotion in middle‐aged to older adults with multiple sclerosis. Journal of the Neurological Sciences, 370, 277–283. 10.1016/j.jns.2016.10.002 27772776

[brb32021-bib-0023] Herold, F. , Wiegel, P. , Scholkmann, F. , Thiers, A. , Hamacher, D. , & Schega, L. (2017). Functional near‐infrared spectroscopy in movement science: A systematic review on cortical activity in postural and walking tasks. Neurophotonics, 4(4), 41403. 10.1117/1.NPh.4.4.041403 PMC5538329 28924563

[brb32021-bib-0024] Herwig, U. , Satrapi, P. , & Schönfeldt‐Lecuona, C. (2003). Using the international 10–20 eeg system for positioning of transcranial magnetic stimulation. Brain Topography, 16(2), 95–99. 10.1023/B:BRAT.0000006333.93597.9d 14977202

[brb32021-bib-0025] Hollman, J. , McDade, E. , & Petersen, R. (2011). Normative spatiotemporal gait parameters in older adults. Gait & Posture, 34(1), 111–118. 10.1016/j.gaitpost.2011.03.024 21531139 PMC3104090

[brb32021-bib-0026] Holtzer, R. , Mahoney, J. , Izzetoglu, M. , Izzetoglu, K. , Onaral, B. , & Verghese, J. (2011). FNIRS study of walking and walking while talking in young and old individuals. The Journals of Gerontology Series A: Biological Sciences and Medical Sciences, 66A(8), 879–887. 10.1093/gerona/glr068 PMC3148759 21593013

[brb32021-bib-0027] Holtzer, R. , Mahoney, J. , Izzetoglu, M. , Wang, C. , England, S. , & Verghese, J. (2015). Online fronto‐cortical control of simple and attention‐demanding locomotion in humans. NeuroImage, 112, 152–159. 10.1016/j.neuroimage.2015.03.002 25765257 PMC4408246

[brb32021-bib-0028] Holtzer, R. , Verghese, J. , Allali, G. , Izzetoglu, M. , Wang, C. , & Mahoney, J. (2016). Neurological gait abnormalities moderate the functional brain signature of the posture first hypothesis. Brain Topography, 29(2), 334–343. 10.1007/s10548-015-0465-z 26613725 PMC4755880

[brb32021-bib-0029] Hsieh, S. , Wu, M. , & Tang, C. (2016). Adaptive strategies for the elderly in inhibiting irrelevant and conflict no‐go trials while performing the go/no‐go task. Frontiers in Aging Neuroscience, 7, 10.3389/fnagi.2015.00243 PMC4701916 26779012

[brb32021-bib-0030] Huxhold, O. , Li, S. , Schmiedek, F. , & Lindenberger, U. (2006). Dual‐tasking postural control: Aging and the effects of cognitive demand in conjunction with focus of attention. Brain Research Bulletin, 69(3), 294–305. 10.1016/j.brainresbull.2006.01.002 16564425

[brb32021-bib-0031] Kahya, M. , Moon, S. , Ranchet, M. , Vukas, R. , Lyons, K. E. , Pahwa, R. , Akinwuntan, A. , & Devos, H. (2019). Brain activity during dual task gait and balance in aging and age‐related neurodegenerative conditions: A systematic review. Experimental Gerontology, 128, 110756. 10.1016/j.exger.2019.110756 31648005 PMC6876748

[brb32021-bib-0032] Kyrdalen, I. , Thingstad, P. , Sandvik, L. , & Ormstad, H. (2019). Associations between gait speed and well‐known fall risk factors among community‐dwelling older adults. Physiotherapy Research International, 24(1), e1743. 10.1002/pri.1743 30198603

[brb32021-bib-0033] Leff, D. , Orihuela‐Espina, F. , Elwell, C. , Athanasiou, T. , Delpy, D. , Darzi, A. W. , & Yang, G. (2011). Assessment of the cerebral cortex during motor task behaviours in adults: A systematic review of functional near infrared spectroscopy (fNIRS) studies. NeuroImage, 54(4), 2922–2936. 10.1016/j.neuroimage.2010.10.058 21029781

[brb32021-bib-0034] Lu, C. , Liu, Y. , Yang, Y. , Wu, Y. , & Wang, R. (2015). Maintaining gait performance by cortical activation during dual‐task interference: A functional near‐infrared spectroscopy study. PLoS One, 10(6), e0129390. 10.1371/journal.pone.0129390 26079605 PMC4469417

[brb32021-bib-0035] Lundin‐Olsson, L. , Nyberg, L. , Gustafson, Y. , Himbert, D. , Seknadji, P. , Karila‐Cohen, D. , Juliard, J. , & Steg, P. (1997). “Stops walking when talking” as a predictor of falls in elderly people. The Lancet, 349, 1. 10.1016/S0140-6736(97)24009-2 9057736

[brb32021-bib-0036] Maidan, I. , Nieuwhof, F. , Bernad‐Elazari, H. , Reelick, M. , Bloem, B. R. , Giladi, N. , Deutsch, J. , Hausdorff, J. , Claassen, J. , & Mirelman, A. (2016). The Role of the frontal lobe in complex walking among patients with parkinson’s disease and healthy older adults: An fNIRS study. Neurorehabilitation and Neural Repair, 30(10), 963–971. 10.1177/1545968316650426 27221042

[brb32021-bib-0037] Marusic, U. , Taube, W. , Morrison, S. A. , Biasutti, L. , Grassi, B. , De Pauw, K. , Meeusen, R. , Pisot, R. , & Ruffieux, J. (2019). Aging effects on prefrontal cortex oxygenation in a posture‐cognition dual‐task: An fNIRS pilot study. European Review of Aging and Physical Activity, 16(1), 2. 10.1186/s11556-018-0209-7 30655911 PMC6329111

[brb32021-bib-0038] Mirelman, A. , Maidan, I. , Bernad‐Elazari, H. , Shustack, S. , Giladi, N. , & Hausdorff, J. (2017). Effects of aging on prefrontal brain activation during challenging walking conditions. Brain and Cognition, 115, 41–46. 10.1016/j.bandc.2017.04.002 28433922

[brb32021-bib-0039] Miyai, I. , Tanabe, H. , Sase, I. , Eda, H. , Oda, I. , Konishi, I. , Tsunazawa, Y. , Suzuki, T. , Yanagida, T. , & Kubota, K. (2001). Cortical mapping of gait in humans: A near‐infrared spectroscopic topography study. NeuroImage, 14(5), 1186–1192. 10.1006/nimg.2001.0905 11697950

[brb32021-bib-0040] Nasreddine, Z. , Phillips, N. , Bédirian, V. , Charbonneau, S. , Whitehead, V. , Collin, I. , Cummings, J. , & Chertkow, H. (2005). The Montreal cognitive assessment, MoCA: A brief screening tool for mild cognitive impairment. Journal of the American Geriatrics Society, 53(4), 695–699. 10.1111/j.1532-5415.2005.53221.x 15817019

[brb32021-bib-0041] Oldfield, R. (1971). The assessment and analysis of handedness: The Edinburgh inventory. Neuropsychologia, 9(1), 97–113. 10.1016/0028-3932(71)90067-4 5146491

[brb32021-bib-0042] Pashler, H. (1994). Dual‐task Interference in Simple Tasks: Data and Theory., 116(2), 25. 10.1037/0033-2909.116.2.220 7972591

[brb32021-bib-0043] Patel, P. , Lamar, M. , & Bhatt, T. (2014). Effect of type of cognitive task and walking speed on cognitive‐motor interference during dual‐task walking. Neuroscience, 260, 140–148. 10.1016/j.neuroscience.2013.12.016 24345478

[brb32021-bib-0044] Pelicioni, P. , Tijsma, M. , Lord, S. , & Menant, J. (2019). Prefrontal cortical activation measured by fNIRS during walking: Effects of age, disease and secondary task. PeerJ, 7, e6833. 10.7717/peerj.6833 31110922 PMC6501770

[brb32021-bib-0045] Pinti, P. , Tachtsidis, I. , Hamilton, A. , Hirsch, J. , Aichelburg, C. , Gilbert, S. , & Burgess, P. (2018). The present and future use of functional near‐infrared spectroscopy (fNIRS) for cognitive neuroscience. Annals of the New York Academy of Sciences, 1464(1), 5–29. 10.1111/nyas.13948 30085354 PMC6367070

[brb32021-bib-0046] Poldrack, R. A. (2005). The neural correlates of motor skill automaticity. Journal of Neuroscience, 25(22), 5356–5364. 10.1523/JNEUROSCI.3880-04.2005 15930384 PMC6725010

[brb32021-bib-0047] Potvin‐Desrochers, A. , Richer, N. , & Lajoie, Y. (2017). Cognitive tasks promote automatization of postural control in young and older adults. Gait & Posture, 57, 40–45. 10.1016/j.gaitpost.2017.05.019 28570861

[brb32021-bib-0048] Quaresima, V. , & Ferrari, M. (2019). A mini‐review on functional near‐infrared spectroscopy (fNIRS): Where do we stand, and where should we go? Photonics, 6(3), 87. 10.3390/photonics6030087

[brb32021-bib-0049] Reuter‐Lorenz, P. , & Park, D. (2014). How does it STAC up? Revisiting the scaffolding theory of aging and cognition. Neuropsychology Review, 24(3), 355–370. 10.1007/s11065-014-9270-9 25143069 PMC4150993

[brb32021-bib-0050] Richer, N. , Saunders, D. , Polskaia, N. , & Lajoie, Y. (2017). The effects of attentional focus and cognitive tasks on postural sway may be the result of automaticity. Gait & Posture, 54, 45–49. 10.1016/j.gaitpost.2017.02.022 28259038

[brb32021-bib-0051] Rosso, A. , Cenciarini, M. , Sparto, P. , Loughlin, P. , Furman, J. , & Huppert, T. (2017). Neuroimaging of an attention demanding dual‐task during dynamic postural control. Gait & Posture, 57, 193–198. 10.1016/j.gaitpost.2017.06.013 28662465 PMC5585862

[brb32021-bib-0053] Schneider, W. , & Shiffrin, R. (1977). Controlled and automatic human information processing: I. detection, search, and attention. Psychological Review, 84(1), 1.

[brb32021-bib-0055] Scholkmann, F. , & Wolf, M. (2013). General equation for the differential pathlength factor of the frontal human head depending on wavelength and age. Journal of Biomedical Optics, 18(10), 105004. 10.1117/1.JBO.18.10.105004 24121731

[brb32021-bib-0056] Shumway‐Cook, A. , Woollacott, M. , Kerns, K. , & Baldwin, M. (1997). The effects of two types of cognitive tasks on postural stability in older adults with and without a history of falls. The Journals of Gerontology Series A: Biological Sciences and Medical Sciences, 52A(4), M232–M240. 10.1093/gerona/52A.4.M232 9224435

[brb32021-bib-0057] Smith, E. , Cusack, T. , & Blake, C. (2016). The effect of a dual task on gait speed in community dwelling older adults: A systematic review and meta‐analysis. Gait & Posture, 44, 250–258. 10.1016/j.gaitpost.2015.12.017 27004667

[brb32021-bib-0058] Sorond, F. , Kiely, D. , Galica, A. , Moscufo, N. , Serrador, J. , Iloputaife, I. , Egorova, S. , Dell’Oglio, E. , Meier, D. S. , Newton, E. , Milberg, W. , Guttmann, C. , & Lipsitz, L. (2011). Neurovascular coupling is impaired in slow walkers: The MOBILIZE Boston Study. Annals of Neurology, 70(2), 213–220. 10.1002/ana.22433 21674588 PMC3152682

[brb32021-bib-0059] Srygley, J. , Mirelman, A. , Herman, T. , Giladi, N. , & Hausdorff, J. (2009). When does walking alter thinking? Age and task associated findings. Brain Research, 1253, 92–99. 10.1016/j.brainres.2008.11.067 19084511 PMC2631095

[brb32021-bib-0060] St‐Amant, G. , Rahman, T. , Polskaia, N. , Fraser, S. , & Lajoie, Y. (2020). Unveilling the cerebral and sensory contributions to automatic postural control during dual‐task standing. Human Movement Science, 70, 102587. 10.1016/j.humov.2020.102587 32217205

[brb32021-bib-0061] Strauss, P. , Sherman, N. , & Spreen, O. (2006). A compendium of neuropsychological tests: Administration, norms, and commentary. Oxford University Press.

[brb32021-bib-0062] Verghese, J. , Wang, C. , Ayers, E. , Izzetoglu, M. , & Holtzer, R. (2017). Brain activation in high‐functioning older adults and falls: Prospective cohort study. Neurology, 88(2), 191–197. 10.1212/WNL.0000000000003421 27927937 PMC5224713

[brb32021-bib-0063] Vermeij, A. , van Beek, A. , Olde Rikkert, M. , Claassen, J. , & Kessels, R. (2012). Effects of aging on cerebral oxygenation during working‐memory performance: A functional near‐infrared spectroscopy study. PLoS One, 7(9), e46210. 10.1371/journal.pone.0046210 23029437 PMC3460859

[brb32021-bib-0064] Wechsler, D. (1981). Wechsler adult intelligence scale‐revised (WAIS‐R). Psychological Corporation.

[brb32021-bib-0065] Woollacott, M. , & Shumway‐Cook, A. (2002). Attention and the control of posture and gait: A review of an emerging area of research. Gait & Posture, 16(1), 1–14. 10.1016/S0966-6362(01)00156-4 12127181

[brb32021-bib-0066] Wu, T. , Kansaku, K. , & Hallett, M. (2004). How Self‐Initiated Memorized Movements Become Automatic: A Functional MRI Study. Journal of Neurophysiology, 91(4), 1690–1698. 10.1152/jn.01052.2003 14645385

[brb32021-bib-0067] Wulf, G. , Shea, C. , & Park, J. (2001). Attention and motor performance: Preferences for and advantages of an external focus. Research Quarterly for Exercise and Sport, 72(4), 335–344. 10.1080/02701367.2001.10608970 11770783

[brb32021-bib-0068] Yesavage, J. , & Sheikh, A. (1986). Geriatric depression scale (GDS). Clinical Gerontologist, 5(1–2), 165–173. 10.1300/J018v05n01_09

[brb32021-bib-0069] Yogev, G. , Hausdorff, J. , & Giladi, N. (2008). The role of executive function and attention in gait. Movement Disorders: Official Journal of the Movement Disorder Society, 23(3), 329–472. 10.1002/mds.21720 18058946 PMC2535903

[brb32021-bib-0070] Yogev‐Seligmann, G. , Hausdorff, J. , & Giladi, N. (2012). Do we always prioritize balance when walking? Towards an integrated model of task prioritization. Movement Disorders, 27(6), 765–770. 10.1002/mds.24963 22419512

